# Anticoagulation clinic drive-up service during COVID-19 pandemic in Qatar

**DOI:** 10.1007/s11239-020-02206-4

**Published:** 2020-07-03

**Authors:** Eman Alhmoud, Osama Abdelsamad, Ezeldin Soaly, Rasha El Enany, Hazem Elewa

**Affiliations:** 1 Al Wakra Hospital, Doha, Qatar; 2 Clinical Pharmacy and Practice Section, College of Pharmacy, QU Health, Qatar University, P.O. Box 2713, Doha, Qatar

**Keywords:** Anticoagulation, Drive-up service, Warfarin, COVID-19

## Abstract

Coronavirus Disease 2019 (COVID-19) is a pandemic affecting many countries worldwide. Given the increasing incidence especially in elderly and individuals with comorbid conditions, it is advised by health authorities to stay home if possible, maintain social distancing and stay away from those who are sick or could be infected. Patients with comorbidities especially cardiovascular disease are at higher risk of getting infected with COVID-19 and have worse prognosis. Among efforts to safely manage warfarin patients during this pandemic, we introduced a hospital drive-up anticoagulation testing service. This service can reduce the risk of exposure of anticoagulation patients to COVID-19 by reducing the contact time with the different personnel at the hospital and by maintaining those patients at a safe distance from others

## Highlights

- Coronavirus Disease 2019 (COVID-19) is a pandemic that has been spreading rapidly worldwide.
- Patients on oral anticoagulation and especially warfarin are typically elderly and have multiple comorbidities which put them at higher risk of getting infected with COVID-19 with poorer prognosis.
- Anticoagulation drive-up service is one of the efforts of healthcare providers to reduce the risk of exposure of anticoagulation patients to COVID-19 by reducing the contact time with the different personnel at the hospital and by maintaining those patients at a safe distance from others.

## Introduction

Coronavirus Disease 2019 (COVID-19) is a pandemic that has been spreading to more than 200 countries worldwide. Given the increasing global spread and disease severity in elderly and individuals with comorbid conditions, Center for Disease Control (CDC) and World Health Organization (WHO) recommend to stay home if possible, maintain social distancing and stay away from those who are sick or could be infected [1–3].

Patients on oral anticoagulation and especially warfarin are typically elderly and have multiple comorbidities. This would put them at high risk for getting infected especially if they have to present to their routine appointments at anticoagulation clinic in an ambulatory care setting or a hospital which has plenty of other patients with various disorders. In a recent study at our clinic, 59% of the patient population had diabetes, 63% were hypertensive and 11% had chronic heart failure [4]. Among our efforts to enhance patients’ safety and minimize contact time at the healthcare facility while maintaining standard clinical care for warfarin patients, we introduced a hospital drive-up anticoagulation testing service at Al Wakra Hospital (AWH) in Qatar.

AWH anticoagulation clinic is one of three specialized anticoagulation clinics in Qatar. It provides pharmacist-managed anticoagulation services since May 2013 for over 300 patients requiring anticoagulation (excluding pediatric and pregnant patients). All patients are managed in-office through face-to-face visits. The patients that are managed at AWH anticoagulation clinic are typically those who live in close proximity to the hospital or have a treating physician at AWH who refers or prefers to have their anticoagulation management to be at the AWH anticoagulation clinic. The clinic operates 5 days/week and is managed by a full-time pharmacist and a full-time nurse. AWH is a general hospital established in 2012 as part of Hamad Medical Corporation (HMC). It provides comprehensive, high quality healthcare to people of all ages, from emergency care, to general medicine and surgery and highly specialized treatments. The hospital has capacity for 325 beds; 248 of which are for general and acute patients and 77 are reserved for critical care, high dependency and burns patients [5].

**Fig. 1:**
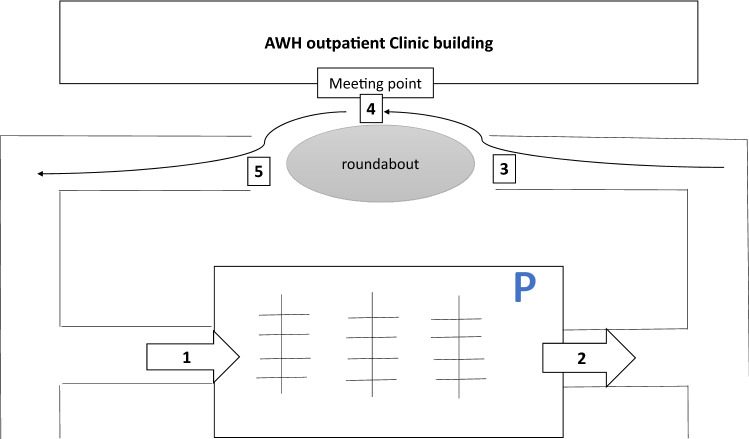
A diagram explaining the flow of the patients to the anticoagulation clinic drive-up service during COVID-19 pandemic. 1—Patient enters to the parking lot and calls the clinic to check-in, 2—Nurse informs the patient to proceed to the drive through designated lane after confirming that it’s clear, 3—Patient proceeds to the testing point, 4—Nurse meets the patient while in the car, confirms identity, scans the patient’s health card, and performs the INR testing, 5—Patient proceeds to the exit unless there is a necessity to proceed to the laboratory or the emergency department or get further instructions from the clinic staff, then proceed back to the parking

AWH drive-up anticoagulation testing service was launched in April, 2020 as part of HMC’s strategy to stay ahead of the pandemic and to assure patients who expressed concerns about visiting the anticoagulation clinic at AWH due to COVID-19 spread. Prior to launching the service, the anticoagulation committee approved the new service and coordinated the implementation among the nursing, finance, registration and pharmacy staffs. Patients were contacted to inform them about the service and educate them about the process including instructions on where to park and the number to call when they arrive for their appointment. Upon patient arrival to the hospital, they park in a designated area very close to the clinic. Then, they call the clinic to inform the staff of their arrival. The anticoagulation nurse asks the patient to drive through a designated lane to the testing spot after it is confirmed to be clear from other cars to avoid accumulation of traffic. The nurse meets the patient while in the car, confirms identity, and scans the patient’s health card. The nurse performs the INR testing using the standard INR point of care testing process used at the clinic (Fig. 1). INR testing results are then transferred wirelessly to the patients’ electronic medical record (Cerner*®*). The pharmacist verifies the INR, and calls the patient to collect all relevant information related to current warfarin dose, medication adherence, changes in diet and medications, incidence of bleeding, thrombosis or other adverse events. Finally, the pharmacist makes recommendation for the new dosing regimen, next follow-up appointment, reinforces education and relays the information to the patient over the phone. Documentation of the visits are performed as usual and are signed-off by the physician. If INR reading is above 5, the nurse would ask the patient to go to the hematology laboratory to recheck the INR before proceeding with the remaining steps of the visit. In case of critical INR values (extreme sub/supra-therapeutic or suspicion of clinical instability), both the pharmacist and the physician in charge would be notified immediately for any necessary actions such as need for bridging, vitamin K or referral to the emergency department. Additionally, HMC has initiated mail delivery service for all patients’ medications to avoid unnecessary contact at the healthcare facilities.

Over a period of 2 months (April 5th–June 11th, 2020), there was a total of 515 visits to the clinic. Compared to the pre-COVID-19 baseline, the reported no-show during this period was 9.5% vs. 6%, which could be due to patients’ fear from leaving home and contracting the infection especially with the increased number of reported COVID-19 cases in the country.

## Discussion

COVID-19 has created a need for innovative services in the various areas of care including anticoagulation. Among the innovations presented at a recent Anticoagulation Forum (AC) Webinar, drive through point of care testing, home testing, extending INR follow-up to a longer (6–12 weeks) versus shorter (4 weeks or less) interval, direct oral anticoagulant (DOAC) switching and telehealth [6]. Extending INR visits gradually to a frequency of up to 12 weeks has been advocated by different guidelines as a strategy of similar safety and efficacy provided patient’s INR has been stable [7, 8]. However, this may not be the most suitable option for patients recently started on warfarin or those with fluctuating INR. Another strategy that is proposed is to switch patients to DOACs. With all the advantages provided with DOACs, their use is not recommended or even contraindicated in certain situations including lactating females, thrombophilia patients, patients having atrial fibrillation with valvular involvement (mitral valve stenosis or mechanical heart valve replacement), and those with advanced renal impairment or on dialysis (except for apixaban) [9, 10]. Thus, we are still left with many of our patient population who need warfarin management and frequent hospital visitation. A drive-through service is deemed to be a safer and a more compassionate option during this time of severe anxiety and uncertainty. It can also improve patients’ adherence with their appointments and satisfaction with the provided care. We believe that this service is feasible and easy to implement. It requires one full-time equivalent (FTE) pharmacist and one FTE nurse (same number of employees that we had in the pre-COVID-19 period). Visits may likely require more time (15–20 min) than the face-to-face visits (10–15 min). However, the daily work load is expected to remain the same since we are reducing the overall number of visits through extending the INR follow-up to a longer (6–12 weeks) versus shorter (4 weeks or less) interval. The physical location of the clinic, the presence of an easy-access testing point, the availability of parking spaces in close proximity to the testing point and the clarity of the phone communication between the clinic and the patient are the most important factors to make this service successful. It is also important to collect data on the quality of anticoagulation management provided to assess the service success and for further implementation. This may include patient satisfaction (through structured surveys), visit frequency and duration, time in therapeutic range, bleeding and thromboembolic events.

Since we started this service the beginning of April, we noticed difficulty communicating with some patients over the phone compared to face-to-face communication. Some patients may get distracted when they are provided with instructions over the phone, while others (especially elderly) may have some hearing problems. It may be important to overcome this, by adding written instructions to those patients via mobile text messaging, or the use of messaging mobile applications (example: WhatsApp, Viber…etc.). Patients not arriving at their scheduled appointments can create overflow of the cars in the drive-through area and may require further coordination. However, we currently ask the patients to go to the parking lot until they receive a call from the clinic to inform them to proceed to the drive-through.

In conclusion, we describe in this communication a recently developed drive-up anticoagulation testing service, which to the best of our knowledge, is the first in the Middle East and North Africa region. This service is one of the efforts of healthcare providers to reduce the risk of exposure of anticoagulation patients to COVID-19 by reducing the contact time with the different personnel at the hospital and by maintaining those patients at a safe distance from others.
